# The language of evidence based medicine: Answers to common questions?

**DOI:** 10.4103/0019-5413.40245

**Published:** 2008

**Authors:** Ryan M Degen, Justin L Hodgins, Mohit Bhandari

**Affiliations:** Division of Orthopaedic Surgery, McMaster University, Hamilton ON, Canada

**Keywords:** Blinding, concealment, definition, evidence-based medicine, randomization, terminology

## Abstract

Evidence based medicine (EBM) is an expanding field that combines clinical intuition with the best available evidence in clinical decision making. The shift to evidence based rationale encourages educating future physicians to formulate appropriate research questions and develop critical appraisal skills that are needed to practice EBM.

This article identifies areas where clinicians may struggle with epidemiological terminology when critically appraising the literature. A review of the relevant terminology encountered in studies that focus on therapy, harm, diagnosis and prognosis can be beneficial to the clinician and are explained within this article.

## INTRODUCTION

The paradigm of medical practice has evolved and with it so must the clinician.^1^ Previously, decision making relied on a solid foundation of clinical expertise, physiologic rationale and traditional medical training. Although clinical instincts remain essential, merely replicating the actions of predecessors is done in a “blind” manner, given that we are oblivious to whether their actions are authoritative (evidence-based) or merely authoritarian (expert opinion).^2^ In light of this, the focus of modern medicine has shifted to rely on empirical evidence to validate clinical decisions, giving rise to “Evidence-Based Medicine” (EBM).

EBM encourages the integration of statistically significant research with clinical experience as well as the patient's unique values and circumstances.^2^ The need for evidence based education has been established in Orthopaedics.^3^ Thus, the emerging orthopaedist must now develop new skills to navigate the constant influx of medical literature and extract the relevant information. Crucial to this skill set is the art of critical appraisal, a process often complicated by epidemiological terminology. The intent of this article is the clarification of language commonly encountered and misinterpreted in EBM.

## HOW DOES EBM DIFFERENTIATE BETWEEN HIGH AND LOWER QUALITY RESEARCH?

It is often helpful when critically appraising an article to first determine the level of evidence [Table 1]. With early identification of an article's level of evidence, inferior resources can quickly be discarded in the interest of time. Evidence from the literature is used to determine the truth of assertion and is organized hierarchically ranging from systematic reviews of randomized trials to unsystematic clinical observations [Table 1]. Levels of evidence for an orthopaedic therapy put highest value on the randomized trial (Level 1 evidence) and least value on surgeon opinion (Level 5 evidence).

**Table 1:** A Hierarchy of study design

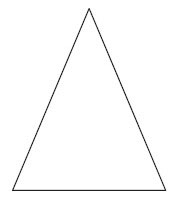	- Systematic reviews of randomized trials
	- Single randomized trial
	- Systematic review of observational studies addressing patient-important outcomes
	- Single observational study addressing patient-important outcomes
	- Unsystematic clinical observations

In a randomized controlled trial (RCT), patients are randomly allocated to either a treatment or control group and followed over time for an outcome of interest. RCTs are the best way to avoid selection or confounding biases and provide an objective basis for quantifying study outcomes. Observational studies differ in that they withdraw inferences from groups of patients based on exposure to certain variables. They do not involve implementing therapies, but rather following groups of patients that have been exposed or retrospectively analyzing patients for an exposure that have experienced the outcome of interest. Since patient or physician preferences can determine whether patients receive a treatment or control, observational studies can have bias.^4^ This concept will be explored in a later section of the article. Clinical observations made by experienced clinicians can provide profound insight into the diagnosis and treatment of illnesses, yet are limited by small sample size and deficiency in human processes of making inferences.^4^ Although the hierarchy is imperfect and exceptions exist, it provides a useful outline to assist physicians in their search for the best available evidence.

## HOW CAN WE CATEGORIZE ORTHOPAEDIC QUESTIONS?

Most clinical questions can be categorized into 4 areas and answered with the results of corresponding studies investigating therapy, harm, diagnosis or prognosis. Therapeutic studies aim to determine the efficacy of various treatments or minimize the occurrence of adverse events. Studies investigating harm identify any potentially harmful agents and their effect on patient function, morbidity and mortality. Diagnostic studies evaluate the ability of an intervention to detect the presence or absence of a specific condition within a population. Lastly, prognostic studies attempt to predict the outcome of the patient's condition. Based on the study's focus, the articles will contain different terminology that must be understood for proper critical appraisal.^4^

## WHAT ARE THE 3 KEY QUESTIONS IN CRITICALLY APPRAISING A RESEARCH ARTICLE?

To determine the integrity of a therapeutic study, 3 important questions must be addressed according to the “Users' Guide to the Medical Literature”:^4^

1. Are the results valid?
2. What are the results?
3. How can the results apply to patient care?

These questions serve as a template from which an article can be appraised and answering each of these questions requires a thorough understanding of the terminology that defines them. The intention of this section is not to describe the proper method of critical appraisal, but instead to define key terms encountered in this process.

## ARE THE RESULTS VALID? RANDOMIZATION, BLINDING, INTENTION TO TREAT AND MORE

To assess the validity of the results, it is important to determine if patients were randomized to their treatment within the study. *Randomization*, commonly referred to as allocation, is a process that uses some method of chance, such as a coin toss, to assign patients to treatment groups.^5^ The random allocation process maximally excludes the difference between two comparison groups as it attempts to neutralize extraneous patient characteristics that may affect the outcome of the treatment provided.^2^,^6^ These patient characteristics are also known as prognostic factors or *determinants of outcome* and include such traits as underlying severity of illness, co-morbid illnesses and patient age.^4^

The next question to consider is whether the randomization process was concealed. Concealment is achieved by taking adequate steps to ensure those responsible for assessing patient eligibility for trial enrolment are kept unaware of whether the patient was allocated to the control or experimental group.^4^

In order to preserve the effects of randomization, it is important that patients are followed and evaluated within the group to which they were initially allocated, regardless of whether they received or completed the intended treatment. This results in an *intention-to-treat analysis*.^4^,^6^

Where possible, it is important to keep the patients, clinicians, outcome assessors and statisticians unaware of the group to which the participant was allocated. This is known as *blinding* and minimizes any differences in patient care other than the intervention under investigation.^4^,^7^ For example, double-blind studies have the patient and either the clinician or researcher blind to the treatment allocation. Blinding the patient eliminates any potential placebo effect. A placebo effect occurs when a patient's expectation of receiving a treatment causes them to either feel or perform better rather than resulting from the action of the treatment itself.^4^

In order for results to be valid, the study must have a sufficiently long and complete follow-up. However this is not always possible since patients can be *lost to follow-up*. The number of patients lost to follow-up can potentially impact the validity of an article. It is important to compare the proportion of patients lost to follow-up to the proportion suffering an adverse event. If information is not available on the prognosis of patients lost to follow up and they represent a significant proportion of the studies' overall population, it is impossible to estimate overall success of the intervention.^8^ Rules of thumb are occasionally outlined, such as the “5 and 20” rule, where if less than 5% of patients are lost to follow-up the effect on the outcome is considered minimal or negligible and if 20% or more of patients are lost to follow-up the validity of the trial is significantly threatened.^2^ These rules are considered to be inaccurate; instead one should consider all patients lost to follow-up as the worst-case scenario and re-analyze the results with those patients' added as failures or adverse events. If this significantly alters the results, then the validity can be considered poor. However, if this does not impact the magnitude of the treatment effect, then the inference made in the trial is secure.^4^

## WHAT ARE THE RESULTS?

When looking at the results of a paper, it is important to determine the magnitude of the treatment effect. This is especially important, because the way in which the results are presented can alter the impact of the intervention making the treatment effect look very small or quite large.^9^ The validity of the results is simpler to interpret when patients experience *dichotomous outcomes*, that is a “yes” or “no” outcome where there is no grey area between.^4^ Examples of such outcomes include implant failure in total joint arthroplasty and pulmonary embolic events in hip fracture patients.

## HOW CAN WE PRESENT DATA FROM A RESEARCH STUDY? RELATIVE RISKS AND RISK REDUCTIONS

Consider the following scenario:

A randomized control trial was performed to determine the efficacy of a prophylactic agent to prevent the occurrence of post-operative deep vein thrombosis (DVT) in patients undergoing ankle fracture repair. In the placebo group, 30% of patients experienced a DVT compared to only 20% of the patients in the treatment group. How might these results be presented?

There are a number of mathematical formulas that can be used in the analysis of study results [Table 2]. The simplest result to demonstrate the improvement in the treatment group is the *absolute risk reduction (ARR) or* the risk difference. This is merely the arithmetic difference between the control event rate (CER) and the experimental event rate (EER).^2^ In the above scenario, the ARR is 30% – 20% = 10%. The impact of treatment can also be expressed using the *relative risk*. This is the risk of an event occurring among patients receiving the experimental treatment relative to that among patients in the control group.^4^ Relative risk is expressed as a ratio, calculated by dividing the EER by the CER.

**Table 2:** Formulas for measurements of therapeutic effect

Measure	Formula
Absolute risk reduction	ARR = (CER) – (EER)
Relative risk	RR = EER/CER
Relative risk reduction	RRR = 1 – RR or = (CER – EER)/CER
Number needed to treat	NNT = 1/ARR × 100
Number needed to harm	NNH = 1/ARI

ARR = absolute risk reduction, CER = control event rate, EER = experimental event rate, RR = relative risk, NNT = number needed to treat, NNH = number needed to harm, ARI = absolute risk increase

Building on this concept, the *relative risk reduction* (RRR) is defined as the difference between the CER and the EER described as a proportion of the CER or calculated simply by subtracting the relative risk from 1.^9^ For the scenario, the RRR would be equal to (30% – 20%)/30% × 100 = 33.33%. In other words, the patients allocated to the treatment arm are 33.33% less likely as those in the control group to develop a DVT post-operatively.

While the relative risk reduction tends to remain constant regardless of the event rate, the absolute risk reduction becomes smaller as the event rate decreases. Thus, the lower the event rate in the control group, the larger the difference between the RRR and ARR.^9^ Consider an additional trial in which the experimental event rate was 2% compared with a control event rate of 3%. When analyzing the results, one would find the RRR to be 33.33%, identical to that of the initial scenario. However, when calculating the ARR, the value is substantially less at only1%. This illustrates the importance of considering event rate in addition to both RRR and ARR. It appeared that the intervention in the additional trial is equally efficacious to the original scenario when looking only at RRR, when in fact its impact is marginal when considering ARR.

## WHAT IS A CONFIDENCE INTERVAL? WHY DOES IT TELL US MORE THAN THE P VALUE?

After determining the magnitude of the treatment effect, the precision should be considered. There are two frequently encountered statistical measures of study precision, *p-values* and *confidence intervals*. The p-value is the probability that the treatment effect can occur in a long run of identical trials as a result of chance alone.^10^ It is commonly agreed that a p-value less than or equal to 0.05 is consistent with statistical significance. For example, consider that the scenario's RRR of 33.33% had a corresponding p value of 0.05. This means that in only 5 trials in a series of 100 could one expect a RRR of 33.33% to be solely due to chance. Although useful, the p-value neglects a piece of information that is essential in clinical decision making - the range over which the effect can possibly occur.^10^ This is addressed with the concept of the confidence interval, which is the range of values within which we can be confident that the true value for the whole population lies.^5^ Traditionally, statisticians report a standard confidence interval of 95%. This means that there is 95% certainty that the true value of the measured variable lies within the stated range.^5^ Intuitive to some, as the sample size of a trial increases so does the number of events and as a result, confidence in the trial result increases as well.^4^ This is best illustrated by the following example taken from the User's Guide to the Medical Literature.^4^

Two trials were conducted in which patients were enrolled and randomly allocated to treatment and control groups to investigate the effect of a prophylactic antibiotic to prevent post-operative infection. The first trial enrolled 100 patients in both the control and treatment groups and recorded 20 infections in the control group and 15 in the treatment group. This equates to a RRR of 25% in the treatment group. However, the perceived RRR can fluctuate considerably given that the large RRR is based on a difference of only 5 infections. Considering this, it is possible by chance that more infections could occur in the treatment group and the intervention could provide no effect or even cause harm. This is evident in the range of the confidence interval, that spans from −38% (infection was 38% more likely to occur in patients receiving the intervention) to 59% (infection was 59% less likely in patients receiving the intervention). In summary, the 95% confidence interval for the RRR ranges from −38 to 59% for RRR and the trial has not convincingly supported that prophylaxis is more beneficial than harmful.

Consider a second trial with 1000 patients enrolled in each of the treatment and control groups. Of these, 200 patients in the control group experienced an infection compared to only 150 patients in the treatment group. As above, this equates to a RRR of 25%. Since this trial size is much larger and has produced more events, the expectation is that the true RRR is more likely to fall around 25% as it is unlikely that the difference of 50 infections occurred by chance alone. In accordance, the confidence interval for this trial is entirely positive ranging from 9% to 41%. Since the confidence interval is greater than zero, this trial is more suggestive of the administration of this intervention as it is more likely to improvement the patients' condition.

## HOW DO WE KNOW IF THE STUDY'S SAMPLE SIZE IS LARGE ENOUGH?

When assessing confidence intervals around a stated relative risk reduction, it is important to determine whether the sample size was large enough and the confidence interval narrow enough to either support or refute the use of the intervention. This can be determined by looking at the upper and lower limits of the stated range. Before considering the limits, the concept of a *minimally important treatment effect* should be introduced. This is simply the smallest amount of benefit that would justify instating the therapy under investigation.^10^ Even if the results of a study are statistically significant which means (exclude a risk reduction of 0), they may not surpass the minimally important treatment effect, as influenced by patient values and features and the treatment would be deemed inappropriate as no benefit would be conferred.^4^

On first inspecting the 95% confidence interval range, it is important to determine if the range of RRR is positive (in support of the treatment) or negative (refuting the treatment). If the confidence interval is found to be predominantly positive in the case of a *positive study*, the next step would be to determine whether the sample size was adequate to support the use of this treatment. This can be determined by looking at the lower limit of the interval and seeing if it lies above the minimally important treatment effect, as previously introduced. If this is true, it can be concluded that sample size was sufficient and the treatment is justified. In the second trial of 1000 patients mentioned above, the lower boundary of the 95% confidence interval was +9% and therefore it can be concluded that the sample size was sufficiently large and the treatment would be beneficial.^4^,^10^

However, if the lower limit is below the minimally important treatment effect threshold, the trial would be deemed to have an inadequate sample size to support use of the intervention. In a *negative study*, the treatment is found to be no better than the control therapy and the confidence interval is predominantly negative. In this case, the upper limit of the range would be inspected and if found to be below zero, the trial can be said to have an adequate sample size and the treatment can be ruled out. However, if the upper limit crosses zero and thus confers some level of improvement, the trial does not have an adequate sample size to dismiss the treatment.^4^,^10^ Consider the first smaller trial of 100 patients, where the RRR range spanned from −38% to +59%. When interpreting this as a negative study, the upper boundary of the RRR crosses into the positive range and the investigators cannot exclude that the treatment may have a positive effect. Therefore, the intervention cannot be excluded as a treatment option.

If authors fail to report the confidence interval around a RRR, the p-value can used to analyze results. If the p-value is precisely 0.05, the lower boundary of the 95% confidence interval has to lie exactly at 0 (a relative risk of 1).^4^ Therefore, the possibility that the treatment has no effect or may cause harm cannot be excluded.^4^ As the p-value falls below 0.05, the lower boundary of the interval for the RRR rises above zero.^4^

## HOW CAN THE RESULTS APPLY TO PATIENT CARE? USING NUMBER NEEDED TO TREAT

In addition to identifying the ARR and RRR, it is crucial to determine how these values will affect patient care. Although a relative risk reduction of 33.33% appears substantial, the impact on the patient in your individual practice may be minimal making the decision to administer the treatment may be difficult. This issue is addressed with the concept of the *number needed to treat* (NNT), which is the number of patients that must receive a particular treatment to prevent one adverse outcome or produce 1 positive outcome.^4^,^9^ The NNT is calculated by dividing 1 by the absolute risk reduction expressed as a percentage. In our scenario, the NNT is 1/33.33 × 100 = 3. Thus, 3 patients need to receive the treatment in order to prevent one adverse event or produce 1 positive outcome. In contrast the *number needed to harm* (NNH) is the number of patients that must receive a particular treatment in order for 1 to experience an adverse effect.^9^ It is calculated by dividing 1 by the *absolute risk increase* (ARI). The ARI is the arithmetic difference between the number of unfavorable outcomes in the treatment and experimental groups.^5^ Both the NNT and NNH are important factors in clinical decision making and should be considered when examining the risks and benefits of the treatment. Patient values and preferences, severity of the outcome prevented, as well as the cost of potential side effects, all play a role in determining at what NNT value therapy should be initiated.^9^

## WHAT IS A COHORT STUDY?

A cohort is a group of individuals that share similar characteristics. *Cohort studies* identify equal sized groups with and without an exposure of interest and follow them forward in time to determine outcomes.^11^ In a *prospective cohort study*, the exposures are identified before study onset and the outcomes of interest identified after a specified follow-up period.^6^ In a *retrospective cohort study*, the outcomes have already occurred before the study was initiated.^6^

For example, a retrospective cohort study may involve surgeons investigating the impact of obesity on the outcome of cemented total hip arthroplasty (THA). Patients with endpoint outcomes would be retrospectively analyzed to determine if their weight had any impact on the long-term outcome of their THA. According to the study performed by Haverkamp *et al.*, THA failure rates were similar among those normal-weight, overweight and obese patients.^12^

Cohort studies are particularly beneficial when looking at infrequent, harmful outcomes when randomization is not feasible. However, the clinician must be cautious about *confounding variables* that can distort the relationship between the study variable and the outcome of interest.^4^ Confounding variables, including age and co-morbid conditions, can give patients a different baseline risk of the target outcome, increasing or decreasing their susceptibility of experiencing a harmful event. A possible source of bias for cohort studies, as well as in RCTs, is a phenomenon known as *surveillance bias*, also called detection bias. In these studies, a control group and treatment group are followed prospectively and there can be a tendency of evaluators to examine the treatment group more closely because of a suspected increase in risk.^4^ Therefore, disease is less likely to go undetected in the treatment versus the control group, generating bias.

Determining *prognosis* requires looking at the possible outcomes of a disease and determining the probability with which they may occur.^4^ This can be done by assessing the results of prognostic studies. *Prognostic studies* enroll patients at a certain stage in the disease process and monitors them forward in time for the frequency and timing of events.^4^ *Prognostic factors* are variables that can either increase or decrease a patients' risk of either a positive or negative outcome.^4^ These factors can be separated into three groups: 1) demographic variables such as age, 2) disease specific variables, such as whether a fracture was intra- or extra-articular and 3) comorbid factors, such as diabetes.

It can be useful to differentiate between prognostic and risk factors, since risk factors often do not alter the outcome of a disease. *Risk factors* are patient characteristics associated with development of disease. For example, obesity is a risk factor for the development of osteoarthritis of the knee, but this is not as significant as the degree of joint space narrowing, a critical factor in determining prognosis.

## WHAT IS A CASE-CONTROL STUDY?

*Case-control studies* are entirely retrospective. Initially, a group of people with a specific outcome are identified and serve as the case group. Subsequently, a group of individuals of similar demographics but without the outcome of interest are identified, representing the control group. Using this design, the groups are analyzed for previous exposures to suspected harmful agents in an attempt to determine if the agents have an association with the target outcome.^4^ For instance, a group of well-healed scaphoid fractures (control) is compared to a group of nonunion scaphoid fractures (cases) to determine if the case-group has an increased proportion of smokers. Case-control studies can be advantageous in investigating outcomes that are rare or slow to develop since the outcome has already occurred.

As with cohort studies, case-control studies are subject to the effects of confounding variables and biases of their own. *Recall bias* occurs when patients who have an adverse outcome are more likely to recall exposure to a harmful substance than those individuals in the control group. This may be explained by a tendency of individuals with an unfavourable outcome to search for a specific variable to attribute their condition to obtain closure. Similarly, *interviewer bias* is generated by greater probing by the investigator into one of the two groups. Since the proportion of patients with an outcome of interest is determined by the investigator, the use of relative risk in study results is not possible in case control studies. Instead, an *odds ratio* is used. This is expressed as the odds of a case-patient being exposed, divided by the odds of a control patient being exposed.^4^

## WHAT IS A CASE SERIES?

Case series (multiple patients) and case reports (single patients) provide no comparison with control groups and simply report on variables thought to be causally linked with the outcome of interest. Although these studies stand lower on the hierarchy of evidence, they have been able to identify substantial adverse events that have changed the standard of treatment. Clinicians are advised to avoid drawing causal relationships from case series and case reports, but instead should use them to generate clinical questions and hypotheses for further study.

## WHAT IS A DIAGNOSTIC STUDY?

Studies that investigate the efficacy of diagnostic tests must use a reference standard or gold standard, as a comparison. The gold standard is an established test that has been shown to be accurate and is well accepted in the medical community as the best available diagnostic test.^4^ When comparing the experimental diagnostic test to the gold standard, it is important that assessors are properly blinded to the reference standard result as it may affect the interpretation of the experimental test.

Often, the indices reported when describing the performance of a diagnostic test are the sensitivity and specificity.^13^ Sensitivity is the proportion of diseased individuals with a positive test result or the “true positives”. Specificity is the proportion of non-diseased individuals with a negative test result or the “true negatives”. To further illustrate this concept, a two column by two column table will be used and should be referenced for calculations [Table 3].

**Table 3:** Diagnostic test table

Diagnostic test result	Disease		Total
		
	Present	Absent	
Positive	a - True positive	b - False positive	(a + b)
Negative	c - False negative	d - True negative	(c + d)
Total	(a + c)	(b + d)	

$\mathrm{Sensitivity}=\frac{a}{\left(a+c\right)}=\frac{\mathrm{TP}}{\left(\mathrm{TP}+\mathrm{FN}\right)}$		$\mathrm{Specificity}=\frac{d}{\left(b+d\right)}=\frac{\mathrm{TN}}{\left(\mathrm{TN}+\mathrm{FP}\right)}$	
$\mathrm{PPV}=\frac{a}{\left(a+b\right)}=\frac{\mathrm{TP}}{\left(\mathrm{TP}+\mathrm{FP}\right)}$		$\mathrm{NPV}=\frac{d}{\left(c+d\right)}=\frac{\mathrm{TN}}{\left(\mathrm{FN}+\mathrm{FN}\right)}$	
$\mathrm{LR for positive test}(\mathrm{LR}+)=\frac{\mathrm{sens}}{1-\mathrm{spec}}=\frac{a/(a+c)}{b/(b+d)}$			
$\mathrm{LR for negative test}(\mathrm{LR}-)=\frac{1-\mathrm{sens}}{\mathrm{spec}}=\frac{c/(a+c)}{d/(b+d)}$			

If a test is highly sensitive and yields a positive result, it is very likely that the patient has the disease. On the other hand, if a highly sensitive test yields a negative result, it is likely to rule out the disease. If a test is highly specific and yields a positive result, the disease can effectively be ruled in. If it yields a negative result, the disease cannot be excluded from the differential diagnosis.

Comparable to these measures, data can also be presented as a positive or negative predictive value. The positive predictive value (PPV) is the proportion of individuals with a positive test result that have the target disease, while the negative predictive *value* (NPV) is the proportion of individuals with a negative test result that do not have the target disease. The PPV and NPV are additional measures of validation for test results. Another variable that is reported when assessing the efficacy of a diagnostic test is the Likelihood Ratio (LR). The LR is the likelihood that a given test result would be expected in a patient with the disorder in question compared with the likelihood that this same result would occur in a patient without the disorder in question.^5^ For example, a LR+ of 20 would mean that a patient with the disease of interest is 20 times more likely to have a positive test result than a patient without the disease of interest. The LR can be calculated by using either the sensitivity and specificity values reported or the raw data provided from the experiment.

A question that often arises after determining the LR is how large or small of a ratio is necessary to either validate or negate a treatment decision.^4^ A LR+ greater than 10 and a LR− less than 0.1 tend to produce the most conclusive changes when using the LR to calculate post-test probability from pretest probability. This step will be discussed shortly.

Before the LR can be used in clinical decision making, the clinician must first determine the probability that the patient has the disease of interest. This estimation is known as the pre-test probability. This step can be problematic since the pre-test probability is often based on a clinician's gut-feeling in accordance with their prior clinical experience. Nevertheless, once the pre-test probability is determined, the clinician can apply the calculated LR to determine post-test probability and then plan their course of action. Using a Fagan nomogram, the conversion from pre-test to post-test probability using the LR can be conducted with a simple straight-edged ruler.

A diagnostic test with a high LR+ is likely to raise the post-test probability of the patient having the disease if the test is positive and would essentially confirm the diagnosis of the disease. A test with a very low LR− is able to lower post-test probability substantially if the test result is negative, enough so to rule the disease out. An ideal test will have both a high LR+ and low LR−, so that both a positive or negative test result will considerably alter the post-test probability and either support further investigation or the initiation of treatment.

How the value of post-test probability will alter physicians' course of action depends on both the *treatment threshold* and *testing threshold*. The treatment threshold is a predetermined value of post-test probability that, if surpassed with the test result, would essentially confirm a diagnosis and the clinician would stop testing and begin treatment. Similarly, the testing threshold is a value of post-test probability below which the clinician would be inclined to administer further testing as no diagnosis is yet evident. Both of these values are not absolute and are subjectively determined based on clinical expertise and situational factors associated with the patient's presentation.

When making choices between diagnostic methods, physicians should incorporate the aforementioned values. Careful examination of the appropriate measures can help to reduce the cost of unnecessary testing and allow the clinician to diagnose disease with more confidence.^4^

## CONCLUSION

Periodic reviews of the terminology used in evidence-based literature can be useful for the emerging clinician as well as the established senior physician. Improving ones understanding of the terminology allows for more accurate and efficient interpretation of the literature. This can enhance ones decision making ability which can ultimately improve patient care.
